# Ageing in relation to skeletal muscle dysfunction: redox homoeostasis to regulation of gene expression

**DOI:** 10.1007/s00335-016-9643-x

**Published:** 2016-05-23

**Authors:** Katarzyna Goljanek-Whysall, Lesley A. Iwanejko, Aphrodite Vasilaki, Vanja Pekovic-Vaughan, Brian McDonagh

**Affiliations:** MRC-Arthritis Research UK Centre for Integrated research into Musculoskeletal Ageing (CIMA), Department of Musculoskeletal Biology, Institute of Ageing and Chronic Disease, University of Liverpool, Liverpool, L7 8XL UK

## Abstract

Ageing is associated with a progressive loss of skeletal muscle mass, quality and function—sarcopenia, associated with reduced independence and quality of life in older generations. A better understanding of the mechanisms, both genetic and epigenetic, underlying this process would help develop therapeutic interventions to prevent, slow down or reverse muscle wasting associated with ageing. Currently, exercise is the only known effective intervention to delay the progression of sarcopenia. The cellular responses that occur in muscle fibres following exercise provide valuable clues to the molecular mechanisms regulating muscle homoeostasis and potentially the progression of sarcopenia. Redox signalling, as a result of endogenous generation of ROS/RNS in response to muscle contractions, has been identified as a crucial regulator for the adaptive responses to exercise, highlighting the redox environment as a potentially core therapeutic approach to maintain muscle homoeostasis during ageing. Further novel and attractive candidates include the manipulation of microRNA expression. MicroRNAs are potent gene regulators involved in the control of healthy and disease-associated biological processes and their therapeutic potential has been researched in the context of various disorders, including ageing-associated muscle wasting. Finally, we discuss the impact of the circadian clock on the regulation of gene expression in skeletal muscle and whether disruption of the peripheral muscle clock affects sarcopenia and altered responses to exercise. Interventions that include modifying altered redox signalling with age and incorporating genetic mechanisms such as circadian- and microRNA-based gene regulation, may offer potential effective treatments against age-associated sarcopenia.

## Introduction

Ageing is a complex multifactorial physiological process involving biochemical and morphological changes at the cellular level that are reflected throughout the organism. Several theories of ageing have emerged over the years, such as mitochondrial dysfunction, accumulation of DNA and oxidative damage, an increase in protein aggregates, as underlying the ageing process. One of the features of ageing and even healthy ageing is a gradual and inevitable loss of skeletal muscle mass and force, sarcopenia, a condition that can significantly impact the quality of life in older adulthood by increasing frailty and potentially reducing independence.

The decline in muscle mass and function in older people appears primarily due to loss of muscle fibres with weakening of the remaining fibres (Marzetti et al. 2009). Moreover, data clearly indicate that in rodents and humans, loss of motor neurons accompanies the loss of muscle fibres (Brown et al. 1988; Butikofer et al. 2011; Campbell et al. 1973; Einsiedel and Luff 1992; Hettwer et al. 2014; Larsson and Ansved 1995; Lexell et al. 1986, 1988; Sakellariou et al. 2014a; Valdez et al. 2010). In humans, Ling et al. have shown that ageing is associated with an increase in motor unit size but a decline in motor unit firing rate in humans (Ling et al. 2009). These data suggest that altered motor unit activation occurs after sarcopenia development (Ling et al. 2009). A study by Piasecki et al. has demonstrated that neuromuscular ageing is associated with loss of motor units and instability of neuromuscular junction (NMJ) transmission in humans however, the loss of motor axons and changes to motor unit potential transmission occur before a clinically relevant loss of muscle mass and function (Piasecki et al. 2015). Whether the changes in the anatomy and function of the NMJ with age play a casual role in sarcopenia development or are a part of the compensatory mechanism aiming at preserving neuromuscular function, remains to be established.

**Fig. 1:**
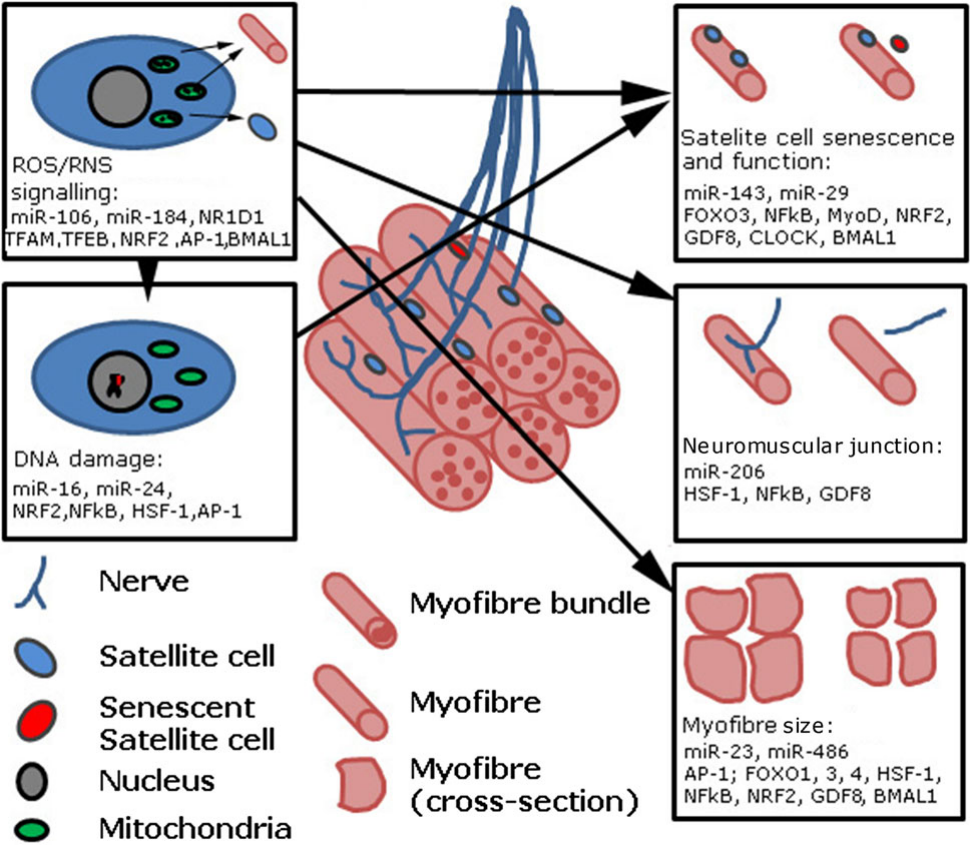
Schematic representation of age-related changes in skeletal muscle and genetic and epigenetic mechanisms associated with these changes

Several studies (Adinolfi et al. 1991; Grover-Johnson and Spencer 1981; Sharma et al. 1980; Verdu et al. 2000) have also shown that neuronal changes and changes at the NMJ play a major role in the age-related loss of muscle mass and function (Delbono 2003). Interestingly, examination of the effects of ageing in peripheral nerves does not reveal an increase in gross oxidative damage similar to the muscle they innervate, but does show an altered redox environment with age (McDonagh et al. 2016). Regeneration of adult skeletal muscle is largely dependent on satellite cells, the skeletal muscle stem cells (Mauro 1961; McCarthy et al. 2011; Morgan and Partridge 2003). The regeneration of skeletal muscle is also reported to be disrupted during ageing (Sousa-Victor et al. 2014) and age-related changes in satellite cell number, their increased susceptibility to apoptosis and impaired ability to proliferate, have been reported in old humans and rodents (Bernet et al. 2014; Brack and Rando 2007; Carlson et al. 2009; Charge and Rudnicki 2004; Collins et al. 2007; Jejurikar et al. 2006; Kadi et al. 2006; Shefer et al. 2010; Snijders et al. 2014; Verdijk et al. 2007). The involvement of satellite cells in age-related muscle wasting remains to be established, as recent data indicate that satellite cell dysfunction may not be a key factor contributing to sarcopenia development (Fry et al. 2015). The main physiological and genetic changes associated with age-related sarcopenia are summarised in Fig. 1.

The disrupted balance between muscle hypertrophy and atrophy, degeneration of NMJ and dysfunction of satellite cells, have all been associated with sarcopenia development. This review discusses the mechanisms regulating disrupted muscle homoeostasis during ageing, focusing on—redox homoeostasis and DNA damage responses, including recent novel developments concerning microRNAs and circadian regulation of gene expression in skeletal muscle and how manipulating these processes could potentially provide a therapeutic opportunity to prevent age-associated sarcopenia.

## Redox associated mechanisms in skeletal muscle ageing

### Redox homoeostasis in skeletal muscle

Regular exercise is increasingly used as a therapeutic strategy to prevent age-related muscular atrophy as well as a number of age-related diseases, including metabolic disorders such as Type 2 diabetes. It is noteworthy that the benefits of exercise may not be equal between young and older people, partially due to the blunted adaptive responses of skeletal muscle in older people (Drummond et al. 2008) (Table 1).

**Table 1:** Key transcription factors associated with maintenance of healthy skeletal muscle and age-associated muscle phenotypes

Transcription factor	Role in muscle	Age-related changes	References
AP-1	Regulator of PGC-1α, interacts with NF-kB and MMP2	Impaired activation with age	Baresic et al. (2014), Hollander et al. (2000), Liu et al. (2010) and Vasilaki et al. (2010)
BMAL/CLOCK	Glucose uptake and oxidation, sarcomeric organisation and fibre-type switching, mitochondrial volume and respiration	Dampened gene expression with age	Andrews et al. (2010) and Dyar et al. (2014)
FOXO1, 3 & 4	Key mediators of catabolic response during atrophy	Decreased activity with age	Furuyama et al. (2002), Milan et al. (2015), Pardo et al. (2008) and Sandri et al. (2004)
HSF-1	Regulates molecular chaperones	Impaired activation with age	Baird et al. (2014) and Vasilaki et al. (2006b)
MyoD	Regulator of muscle-specific target genes involved in myogenic differentiation	Expression upregulated during ageing	Dedkov et al. (2003)
NFκB	Role in muscle atrophy during fasting	Constitutively active in old and dysregulated activation after exercise	Lee and Goldberg (2015) and Vasilaki et al. (2010)
Nrf2	Master regulator of antioxidant proteins	Impaired activity and contributes to muscle atrophy, muscle progenitor function	Miller et al. (2012), Safdar et al. (2010) and Narasimhan et al. (2014)
Myostatin (GDF8); Smad2/3	Regulation of muscle hypertrophy	Not clear due to complex post-translational modifications	Sartori et al. (2009) and White and LeBrasseur (2014)
TFAM	Maintenance and organisation of mtDNA	Impairment of pathway with age, closely related to activity	Bori et al. (2012), Larsson et al. (1998) and Picca et al. (2014)
TFEB	Coordinates expression of autophagic and lysosomal genes	Key regulator of autophagy and modulation of longevity	Lapierre et al. (2015, 2015)
REV-ERBα (NR1D1)	Mitochondrial biogenesis, autophagy and exercise tolerance	Not investigated	Woldt et al. (2013)

The metabolic and structural changes induced by exercise ultimately affect the contractile properties of the muscle fibres and as contracting muscles generate ROS/RNS, these signalling molecules play crucial roles in the adaptive response to exercise and muscle atrophy during ageing (Table 2, Powers and Jackson 2008). ROS/RNS generate unique signalling cascades that are essential in skeletal muscle contraction and adaptation, but also play a role in a wide array of cell processes including cell proliferation, immune response and antioxidant defences. The age and fitness of the individual as well as the intensity and duration of muscle contractions will have an effect on both the levels and type of ROS/RNS generated. Dysregulated ROS/RNS homoeostasis or signalling responses have been reported in a wide variety of human skeletal muscle disorders, as well as many metabolic diseases including cancers (Mates et al. 2008), neurodegeneration (Calabrese et al. 2006), obesity (O’Neill et al. 2013) and diabetes (Gil-del Valle et al. 2005). Key proteins and transcription factors activated in response to muscle contraction can be directly or indirectly regulated by the concentrations of ROS/RNS e.g. NFκB (Ji et al. 2004), sarcoplasmic–endoplasmic reticulum Ca^2+^ ATPase (SERCA) (Tong et al. 2010) and ryanodine receptor 1 (RyR1) (Sun et al. 2013).

**Table 2:** Selected redox homoeostasis processes associated with exercise and ageing proposed to play an important role in muscle homoeostasis

Process	Ageing-associated consequences	Proposed mechanism
Muscle contraction	Increased oxidation of contractile proteins; disrupted fatty acid oxidation due to accumulation of dysfunctional mitochondria	Changes in mitochondrial activity (Gerhart-Hines et al. 2007; Carnio et al. 2014)
	Disrupted autophagy resulting in defective regeneration and myofibre atrophy	Activation of autophagy (Sandri 2010; Tang and Rando 2014; Garcia-Prat et al. 2016)
Neuromuscular function	Disrupted redox balance resulting in myofibre atrophy, NMJ degeneration	SOD1 activity (Sakellariou et al. 2014a)
Satellite cell function	Disrupted protection against excessive ROS and DNA damage resulting in disrupted quiescence of satellite cells and their niche resulting in defective regeneration	Activity of antioxidant enzymes (Pallafacchina et al. 2010)
Senescence	Oxidative stress and deterioration in muscle regeneration satellite cell senescence	ROS production in satellite cells (Garcia-Prat et al. 2016)

### Sources of ROS/RNS in skeletal muscle

There are a number of potential endogenous sources of ROS/RNS in skeletal muscle. Recent studies indicate that cytoplasmic generation, rather than electron leakage from the electron transport chain, are primarily responsible for the increase in ROS/RNS generation within healthy skeletal muscle following contractions (Pearson et al. 2014; Sakellariou et al. 2013, 2014b; Ward et al. 2013). As ROS/RNS are too reactive to produce a signalling effect by diffusion throughout the cell, it is now considered that they produce an effect localised to their site of generation (Poole 2015). In relation to skeletal muscle, two known endogenous producers of ROS/RNS are the NAD(P)H oxidase (NOX) family and nitric oxide synthases (NOS) and they predominantly generate superoxide (O_2_^·−^) and nitric oxide (NO), respectively. Isoforms of both these proteins are in an ideal position to control redox mechanisms in muscle as they are known to co-localise within the T-tubule (Stamler and Meissner 2001; Ward et al. 2013). Neuronal and endothelial NOS (nNOS and eNOS) contain a calmodulin binding site making them sensitive to Ca^2+^ release and muscle contractions (Stamler and Meissner 2001). Indeed NO and O_2_^·−^ produced by NOS and NOX may react and form another redox oxidant, peroxynitrite (ONOO^−^), these species of ROS/RNS have different chemical reactivities and kinetics, resulting in distinct post-translational modifications on target proteins with potentially further downstream redox effects. Ageing itself could result in different species and concentrations of ROS/RNS generated as a result of muscle contractions. This could potentially have a significant effect on downstream protein targets and their activity with further far reaching effects on the molecular responses to exercise.

### Redox regulation of transcription factors in skeletal muscle

Skeletal muscle can respond rapidly to exercise and other environmental changes by modifying the expression of genes involved in the regulation of muscle structure, mass and energy metabolism. The ROS/RNS generated during contractile activity have been reported to lead to the activation of a number of redox-regulated transcription factors, including NFκB, AP-1, HSF-1 and NRF2 (Table 1) with a subsequent increased expression of regulatory enzymes in both pro- and anti-inflammatory processes depending on the level of ROS/RNS generated and the transcription factor activated (Ji et al. 2004; Vasilaki et al. 2006a). For instance, it has been shown that NFκB is constitutively activated in muscle tissue from old mice while in adult mice it is activated only after intense muscle stimulation (Vasilaki et al. 2010).

Redox-regulated processes coupled with muscle contractions are reported to stimulate the expression of genes associated with autophagy and cellular longevity, especially the regulation of the helix-loop-helix transcription factor TFEB and the forkhead (FOXO) transcription factors (reviewed in Lapierre et al. 2015). TFEB regulates the transcription of genes involved in autophagic induction, phagophore formation and degradation, while its phosphorylation is mTOR dependent and prevents its translocation to the nucleus (Settembre et al. 2011). FOXO transcription factors also regulate many of the genes involved in the autophagy core machinery, glucose metabolism, the antioxidant response and can directly interact with AMPK, thus the activation of these transcription factors is highly dependent on the nutrient-sensing pathways and energy availability. Changes in ROS/RNS generation and subsequently redox signalling have an effect on a wide variety of cellular control points including, hypoxia-inducible factor 1α (HIF-1α), activator protein 1 complex (AP-1), NFκB, the sirtuin deacetylase enzymes, mitochondrial biogenesis and can have a wide-ranging influence on the cellular energy flux. A number of recent studies have linked the redox state of the thiol peroxidases peroxiredoxins (Prxs) and H_2_O_2_ signalling to the activation of specific cellular pathways e.g. Prx1 activation of the transcription factor Ask1 (Jarvis et al. 2012) and Prx2 forms a redox relay with the transcription factor STAT3, generating oligomers with modified transcriptional activity (Sobotta et al. 2015). Similarly, the FOXO3 transcription factor has been shown to require the formation of intermolecular disulphides with the nuclear receptors, importin 7 and 8, for ROS-induced nuclear translocation and activity (Putker et al. 2015). Redox signalling mediated by disulphide exchange on redox-sensitive proteins and receptors at physiologically relevant concentrations of H_2_O_2_, would allow rapid activation and/or translocation to the nucleus of transcription factors in muscle fibres in response to contractile activity.

### Altered redox homoeostasis and skeletal muscle ageing

Ageing muscle has as an altered redox response with subsequent physiological and biochemical effects on the cytoskeleton, mitochondria, calcium signalling and sequestration (Egan and Zierath 2013; Labunskyy and Gladyshev 2012; McDonagh et al. 2014). Older individuals that have maintained an exercise regime throughout their lifetime have been reported to have preserved biochemical markers of muscle fitness [mtDNA, peroxisome proliferator-activated receptor coactivator 1α (PGC-1α)] that are comparable to younger individuals (Cobley et al. 2012). A recent study highlighted the plasticity of skeletal muscle by reversing the biochemical effects of ageing with NAD^+^ treatment (Gomes et al. 2013). Acute bouts of strenuous exercise however, can result in muscle damage that contrasts to the adaptive improvement that is achieved from exercise as a therapeutic agent (Aoi et al. 2013). Muscle damaging effects of strenuous exercise can result in a dysregulated redox response within the muscle compared with the adaptive responses and benefits of a transient increase in ROS/RNS. It is also worth mentioning that although exercise ameliorates, it does not prevent the progressive loss of muscle strength that occurs in the elderly, suggesting that blunted adaptive responses to exercise, potentially through redox-mediated mechanisms, occur with ageing.

Acute exercise induces autophagy in both skeletal and cardiac muscle (He et al. 2012). In relatively long-lived skeletal muscle fibres the adaptive response to exercise requires the degradation of cellular components, allowing the muscle fibre to rebuild and respond to repetitive bouts of exercise. Interestingly, myofibrillar components of muscle are mainly degraded by the ubiquitin–proteasome system while dysfunctional organelles and protein aggregates are degraded through autophagy. Autophagy is a key house-keeping mechanism where the interplay between autophagy and cellular damage is finely balanced and can be the difference between beneficial adaptation, cell death or accumulation of damaged cellular organelles (He et al. 2012). Indeed, excessive autophagy can be detrimental in skeletal muscle resulting in muscle wasting and has been reported in a number of cancers, cachexia and dystrophies (Vainshtein et al. 2014). Reduced mitochondrial turnover and decreased autophagy have been associated with a number of chronic conditions, including ageing in skeletal muscle, with increased myosin and actin oxidation resulting in decreased force due to reduced sliding properties in the fibre (Romanello and Sandri 2015). Recent studies have demonstrated that inhibition of autophagy can increase mitochondrial damage not only within the muscle but also in the NMJ and to ultimately affect animal lifespan (Carnio et al. 2014). Accumulation of damaged or dysfunctional mitochondria has been demonstrated to increase the oxidation of contractile proteins in muscle whilst enhancing autophagy can contribute to longevity (Carnio et al. 2014), with potentially important consequences in the elderly for the maintenance of muscle mass and function. Acute exercise can cause rapid changes in mRNA levels within a cell but changes in protein content generally occur over a relatively longer period.

## DNA damage and skeletal muscle

### Markers of DNA damage

As with all theories concerning the mechanism of skeletal muscle ageing, it is difficult to definitively assign a causal role to DNA damage and repair in the process. However, without doubt, DNA damage has the potential to be deleterious either as a direct consequence of the DNA damage itself or as a downstream effect of the DNA repair processes. Not surprisingly perhaps, excessive ROS/RNS generation is the primary source of DNA damage in skeletal muscle. There are numerous DNA lesions that can occur as a consequence of the excessive generation of ROS/RNS including abasic sites, strand breaks and >100 base modifications, which if unrepaired, lead to mutations and genomic instability, especially in replicating DNA such as mtDNA, that play essential roles in ageing.

Of the four nucleotides, guanine has the lowest oxidation potential and is therefore most vulnerable to oxidative modifications (Steenken and Jovanovic 1997). The most abundant, and most frequently studied base modification is 8-oxo-7,8-dihydroguanine (8-oxoG), which can result from direct attack on the DNA or incorporation of an oxidised base from the nucleotide pool. 8-oxoG has the potential to be mutagenic because it can adopt the *syn* configuration and during DNA replication forming a Hoogsteen pair with adenine (in the *anti*-configuration) resulting in a G:C to T:A transversion mutation (Cheng et al. 1992). Particularly relevant is the observation that 8-oxoG may affect transcription factor binding in G4-rich promoters e.g. binding of Sp1 (Clark et al. 2012), which has been shown to promote myoblast differentiation (Yuan et al. 2013) and the expression of the anti-inflammatory NFκB p50 subunit (Hailer-Morrison et al. 2003). 8-oxoG can also block transcription elongation via RNA polymerase II (Kuraoka et al. 2003) or result in the production of mutant transcripts (Bregeon et al. 2009). There are only a few studies of 8-nitrodG primarily because it is unstable. However, a recent study (Bhamra et al. 2012) has provided evidence that 8-nitrodG has the potential to not only mispair with adenine leading from G:C to T:A transversion mutations, but may also stall replication forks resulting in single strand breaks (SSBs) and gaps which can convert to double strand breaks (DSB). 8-oxoG may also lead to increased DNA damage such as further oxidation products (Morikawa et al. 2014) and the development of SSBs via the base excision repair (BER) pathways which can either remove the 8-oxoG (Caglayan and Wilson 2015) or remove the mispaired adenine (Sheng et al. 2012). In addition to the effects of BER, SSBs can also be created via direct ROS attack on DNA. Furthermore, if two SSBs are close together or they occur in DNA which is being replicated e.g. mtDNA or proliferating satellite cells, the SSBs can be converted into DSBs which are lethal if not repaired (O’Driscoll and Jeggo 2008). Interestingly, elevated levels of 8-oxoG were detected in the *Vastus lateralis* of aged individuals compared to young subjects potentially linking nDNA damage and skeletal muscle dysfunction (Radak et al. 2011). Interestingly, the levels of 8-oxoG in old sedentary subjects were higher compared to old active subjects, indicating that physical activity may confer a genoprotective effect.

### DNA damage and skeletal muscle

The cell fate following DNA damage—DNA repair, cell cycle arrest, apoptosis, senescence or necroptosis (Gebel et al. 2013; Vicencio et al. 2008), is dependent on a range of factors including the cell type e.g. satellite cells, muscle fibres or motor neurons, the type, extent, site (e.g. regulatory v coding; nuclear v mitochondrial), and time of the DNA damage and the health, age and exercise status of the individual. Skeletal muscle satellite cells are key components required for muscle repair following muscle injury. The normally quiescent satellite cells are stimulated to enter the cell cycle following damage to the muscle. Following activation, there is either commitment of the progeny to the production of myoblasts, which then fuse with the muscle fibre, or there is self-renewal of the satellite cells in their niche. It is therefore essential that the genome integrity of satellite cells is maintained either by protection from DNA damage, removal of damaged cells and/or enhanced repair. All of these processes appear to be involved in preventing genomic instability in stem cells but with age these processes begin to fail, leading to genome-wide accumulation of passenger mutations, reduced replicative capacity and altered progenitor cells, ultimately resulting in defective tissue maintenance (Burkhalter et al. 2015). Satellite cells have high transcript levels for genes involved in the protection against excessive ROS (Pallafacchina et al. 2010) and their niche helps protect them from oxidative stress-induced damage. Satellite cells are also able to repair DSBs more efficiently than their differentiated progeny, myoblasts and differentiated myonuclei (Vahidi Ferdousi et al. 2014). The ability of satellite cells to regenerate declines with age and although there may be a slight reduction in repair capacity, there does not seem to be any obvious age-related difference in the accumulation of DNA damage especially DSBs (Cousin et al. 2013). Proliferating muscle stem cells appear to have more DSBs than satellite cells but unlike satellite cells they use both c-NHEJ and HRR to repair these breaks (Vahidi Ferdousi et al. 2014). Myoblasts also have higher levels of poly-ADP-ribose-polymerase 1 (PARP1) compared to differentiated cells which correlates with an increase in resistance to oxidative stress in the myotubes (Olah et al. 2015).

There is considerable evidence linking DNA damage (in mitochondrial and nuclear genomes) and defects in DNA repair mechanisms to neurodegenerative diseases (Iyama and Wilson 2013), although there have been few studies on the effects of DNA damage on the viability of motor neurones. However, a recent study (Chu et al. 2014) has demonstrated that H_2_O_2_ causes a downregulation of APE1 (a key BER protein required for the repair of 8-oxoG and abasic sites in both nuclear and mtDNA in spinal motor neurons). In addition, because APE1 is also a redox responsive protein, this downregulation increased the sensitivity of the motor neurons to oxidative stress and likelihood of cell death. Further studies are required to investigate the role(s) of oxidative stress-induced DNA damage and downregulation of APE1 in motor neurons in muscle ageing.

There is also growing evidence linking the protective effects of exercise against DNA damage and increase in repair mechanisms during ageing. However, the consequences of damage and repair may be more complex than mutations affecting genomic stability or cell fates such as apoptosis, and more work is required to understand their relevance to muscle ageing.

## Post-transcriptional regulation of age-related muscle loss in skeletal muscle

### MicroRNAs and skeletal muscle ageing

MicroRNAs (miRNAs, miRs) are short, non-coding RNAs that regulate gene expression at the post-transcriptional level, they are implicated in many biological processes, including muscle function and disease (Goljanek-Whysall et al. 2012). miRNAs are predicted to target two-thirds of the human genome (Friedman et al. 2009), affecting the mRNA and protein content while providing a robust mechanism of responding to changes within the cell or the surrounding environment. Muscle-specific miRNAs are crucial regulators of skeletal muscle function (Brown and Goljanek-Whysall 2015; Goljanek-Whysall et al. 2012; Soriano-Arroquia et al. 2016a) and differential expression of miRNAs are associated with muscle disorders, such as muscular dystrophy (Eisenberg et al. 2007; Elia et al. 2009; Soares et al. 2014; Williams et al. 2009). The ability of miRNAs to target many genes regulating specific physiological processes, their conservation among species, as well as the ability of manipulating the expression of miRNAs in vitro and in vivo, has contributed to the growing interest in miRNAs as potential therapeutic agents. Although the involvement of specific miRNAs in the development of sarcopenia remains unclear, key physiological processes affected by ageing including both hypertrophy and atrophy, as well as muscle regeneration, are regulated by miRNAs, and likely important regulators of age-related decline in muscle mass and function.

Skeletal muscle senescence has also been shown to be regulated by miR-29. miR-29 expression is upregulated in skeletal muscle of mice during ageing. miR-29 has been shown to regulate the expression of p85alpha, IGF-1 and B-myb (Hu et al. 2014). Upregulation of miR-29 expression was associated with impaired proliferation of muscle progenitor cells and their senescence. Moreover, miR-29 overexpression in vivo, in muscles of adult mice, led to a senescent phenotype of muscle and muscle atrophy (Hu et al. 2014).

Satellite cell-specific knock out of the enzyme Dicer leads to defective muscle regeneration and premature muscle wasting suggesting that miRNAs may play a pivotal role in maintaining satellite cell potential in adulthood (Cheung et al. 2012). Our work has shown that miR-143 may be associated with, at least to some degree, controlling satellite cell senescence via regulating the expression of IGFBP5 (insulin growth factor-binding protein 5) (Soriano-Arroquia et al. 2016b). Interestingly, age-related changes in miR-143 expression in satellite cells appear to be a part of a compensatory mechanisms aiming at ameliorating age-related changes in satellite cell function rather than a causative mechanisms responsible for these changes; this keeps in with previously published role of miRNA-206 in ALS (Williams et al. 2009).

### MicroRNAs and the neuromuscular junction

NMJ deterioration is an important physiological aspect of ageing associated with muscle atrophy. NMJ formation is regulated by miRNAs—mice deficient of Dicer, an enzyme required for the maturation of most miRNAs, show impaired reinnervation following nerve injury (Valdez et al. 2014). Using the amyotrophic lateral sclerosis (ALS) mouse model, characterised by deterioration of NMJs and muscle atrophy, Williams et al. have demonstrated that muscle-enriched miR-206 (myomiR) is a key regulator of muscle–nerve interactions and is required for efficient regeneration of NMJ after nerve injury by regulating *Hdac4* (Williams et al. 2009). Interestingly, another myomiR, miR-133b, expression of which is concentrated near NMJs and induced following denervation, has been shown not to play a role in NMJ maintenance and reinnervation (Valdez et al. 2014) suggesting that miR-206 may be the key regulator of NMJ deterioration.

### Redox homoeostasis and microRNAs

One of the key mechanisms associated with muscle ageing is disrupted redox homoeostasis (Gianni et al. 2004). miRNAs may be linked to the redox status of the muscle through post-transcriptional regulation of key enzymes involved in ROS/RNS signalling (Togliatto et al. 2013). Overexpression of miR-106b led to mitochondrial dysfunction and insulin resistance in C2C12 myotubes by targeting the expression of mitofusin-2 (Mfn2), as well as PGC-1α (Zhang et al. 2013). Interestingly, ROS signalling has also been shown to regulate not only the expression of miRNAs controlling important aspects of skeletal muscle homoeostasis, but also miRNA function through modifications of the mature microRNAs (Wang et al. 2015). Oxidative modification of mature miR-184 has been shown to modify miRNA:target interactions. Wang et al. have demonstrated that oxidative modification of miR-184 is associated with miR-184 targeting its non-native targets: Bcl-xL and Bcl-w, effectively leading to apoptosis initiation. As redox homoeostasis is disrupted in muscle during ageing and considering the rather long half-life of a vast number of miRNAs (Olejniczak et al. 2013), oxidative modification of miRNAs could affect interactions with their target genes and be one of the mechanisms associated with age-related loss of muscle mass and function.

DNA damage itself has also been shown to modulate miRNA processing and maturation therefore affecting miRNA:target interactions (Hu and Gatti 2011). The expression of several miRNAs associated with controlling muscle and satellite cell function (Cheung et al. 2012; Soriano-Arroquia et al. 2016b) e.g. miR-16, miR-143, are upregulated following irradiation-induced DNA damage through a p53-dependent modification of the Drosha/DGCR8 complex associated with miRNA maturation (Suzuki et al. 2009). Another study by Boominathan 2010 has demonstrated the regulation of miRNA processing by DNA damage response factors, through controlling of miRNA processing machinery (Boominathan 2010). miRNAs are also emerging as a novel regulatory layer of DNA damage response (DDR) regulation (Hu and Gatti 2011). For example, miR-24 has been shown to downregulate the expression of histone variant γH2AX, the sensor of the double strand break in DDR (Lal et al. 2009), whereas miR-21, has been shown to participate in the DDR by controlling the expression of CDC25a, a cell cycle regulator (Wang et al. 2009). Interestingly, miR-21 has also been shown to drive the fibrosis of skeletal muscle (Ardite et al. 2012).

Exercise, the only current effective intervention against muscle wasting, has also been associated with changes in miRNA expression in skeletal muscle. For example, Nielsen et al. (2010) have shown that miR-206, miR-133 (myomiRs) expression is modulated following acute and endurance exercise in humans and myomiR levels decrease to a new steady-state level following an inactivity period (Nielsen et al. 2010). Moreover, an acute bout of resistance exercise has been shown to be associated with downregulation of miR-1 in human muscle (McCarthy and Esser 2007), suggesting that changes in miRNA expression may be an important mechanism associated with muscle adaptive responses. Comparison of miRNA expression in the skeletal muscle from young and old men before and after acute resistance exercise, revealed that changes in miRNA responses following exercise were absent in the older group, suggesting the involvement of miRNAs in regulating skeletal muscle adaptive response to contractions, which is defective in the muscles of older people (Rivas et al. 2014).

Together, these data indicate that miRNA regulation is an integral part of skeletal muscle ageing. Understanding the disrupted miRNA:target interactions in sarcopenia development may provide novel therapeutic avenues against age-related loss of muscle mass and function.

## Circadian regulation in skeletal muscle

### Circadian rhythms and the molecular clock

Circadian rhythms are the ~24 h biological cycles in physiology, behaviour and metabolism that function to temporally organise and prepare an organism for daily environmental changes (Hastings et al. 2003; Reppert and Weaver 2002). They are highly evolutionarily conserved from single-cell organisms to mammals (Loudon 2012). In mammals, the circadian pacemaker lies in the suprachiasmatic nucleus (SCN), located in the anterior hypothalamus, and is referred to as the central clock (Dibner et al. 2010). It is entrained by light/dark cycles and it imposes synchronicity to peripheral organs such as skeletal muscle (Yamazaki et al. 2002). Recent studies have pointed out the importance of autonomous peripheral clocks existing in every tissue, which respond to external cues (called ‘Zeitgebers’), such as feeding or exercise, and therefore function in the environmental adaptation of organisms (Schroeder et al. 2012; Wolff and Esser 2012). In skeletal muscle, >800 genes are expressed in a circadian pattern (Schroder and Esser 2013), and these genes participate in a wide range of functions including myogenesis, transcription and metabolism (McCarthy et al. 2007; Miller et al. 2007).

The circadian oscillations in physiology and metabolism are governed by the molecular clock, a transcriptional/translational feedback mechanism that is present in virtually all cells of an organism (Ko and Takahashi 2006). In mammals, the molecular clock components comprising the positive arm of the core clock are two members of the PAS-bHLH family of transcription factors, CLOCK (Circadian locomotor output control kaput) and BMAL1 (Brain muscle arnt-like1). The CLOCK:BMAL1 heterodimer activates transcription of core clock genes *Period* (*Per1*, *Per2* and *Per3*) and *Cryptochrome* (*Cry1* and *Cry2*) by binding to E-box (CACGTG) sequences in the regulatory region of these genes. The CRY and PER proteins constitute the negative arm of the core molecular clock by forming multimers that inhibit CLOCK:BMAL1 transcriptional activity upon translocation to the nucleus. Additional components to the core molecular clock family include the orphan nuclear receptors RORA (RAR-related orphan receptor-α) and REV-ERB α/β (NR1D1/2), which function by activating (ROR) or repressing (REV-ERB) *Bmal1* transcription. In addition to their role in timekeeping, components of the core clock such as BMAL1 and CLOCK have also been shown to transcriptionally regulate the expression of genes that do not function in timekeeping and these genes are designated as clock-controlled genes (CCGs) (Gossan et al. 2013; Panda et al. 2002). Some of the direct CCGs in specific tissues, such as skeletal muscle, often encode transcription factors/co-activators (MyoD1, PGC1α) (Andrews et al. 2010; Liu et al. 2007; Zhang et al. 2012), genes that control rate-limiting steps in cell physiology or genes regulating specific muscle functions such as contractility, genes involved in calcium handling, metabolism (e.g. ATP synthases) (McCarthy et al. 2007) and cell signalling (e.g. Wnt) (Chatterjee et al. 2013; Burke et al. 1996).

### Post-transcriptional regulation of the molecular clock

Emerging evidence indicates an important role for modulation of the molecular clock by the post-transcriptional regulation, from splicing, polyadenylation and mRNA stability to translation and non-coding functions exemplified by miRNAs (Kojima et al. 2011; Staiger and Green 2011). This level of regulation affects all aspects of the circadian system, from the core timing mechanism and input pathways that synchronise clocks to the environment and output pathways that control overt rhythmicity, thus enhancing robustness as well as the ability to adapt to different environments (Hansen et al. 2011; Kojima et al. 2011). Recent studies indicate a special role for miR-132 and miR-219 in mediating entrainment of the circadian clock in mice (Alvarez-Saavedra et al. 2011; Cheng et al. 2007). These miRNAs are regulated by the circadian and light-induced transcription factor CREB. miR-219, which is directly regulated by the CLOCK:BMAL1 heterodimer, affects the circadian pacemaker. In contrast, miR-132, appears independent of CLOCK:BMAL1, but displays circadian oscillations in the SCN that are dependent on the negative clock regulators CRY1 and CRY2. Moreover, miR-132 can also modulate the negative arm of the circadian clock as well as the responses of the molecular clock machinery to light. In addition to miRNAs, the timing of the molecular clock can be modulated by post-translational mechanisms through phosphorylation, acetylation and/or ubiquitination pathways (Lee et al. 2001; Li et al. 2013). These modifications impact the stability and/or translocation of core molecular clock components and often affect the periodicity of oscillations. Further work is needed to decipher the role of post-transcriptional mechanisms and miRNAs in peripheral tissue clocks such as skeletal muscle.

### Models of circadian clock disruption and skeletal muscle ageing

The importance of circadian rhythms for skeletal muscle is evidenced by the skeletal muscle phenotypes observed in mouse models of genetic clock disruption (reviewed in Mayeuf-Louchart et al. 2015). Global loss of the core clock gene *Bmal1* results in accelerated sarcopenic phenotypes, including severe muscle degeneration (reduced total mass), fibre-type shifts, decreased mitochondrial volume and respiration (with increased respiratory uncoupling), altered sarcomeric structure and reduced force production at both the whole-muscle and single-fibre level (Kondratov et al. 2006; Andrews et al. 2010; Dyar et al. 2014). Moreover, another model of genetic disruption of the positive arm of the clock, *Clock*^*Δ19*^ mutant mice, display a reduction in skeletal muscle force and decreased exercise tolerance, which is associated with decreased mitochondrial content/activity and perturbations of muscle architecture involving decreased expression of structural proteins (Hodge et al. 2015; Jeong et al. 2015; Kondratov et al. 2006). However, these perturbations do not affect the adaptation of *Clock*^*Δ19*^ mice to chronic exercise (Pastore and Hood 2013). In addition, *Bmal1*^*KO*^ mice show detrimental effects on metabolic health, including impaired glucose tolerance and insulin sensitivity (Kondratov et al. 2006). Mice mutated for *Per2*, a component of the negative arm of the core clock, also have lower running endurance and locomotor performances due to an increased reliance on glycolytic anaerobic metabolism rather than an alteration of muscle contractility (Bae et al. 2006; Zheng et al. 1999). Recent work has also demonstrated that *Rev*-*erbα* deficiency (a component of the stabilising loop) results in lower exercise capacities, due to impaired mitochondrial biogenesis and an increase in autophagy in skeletal muscle (Woldt et al. 2013). Rescuing the expression of *Bmal1* specifically in the skeletal muscle of global *Bmal1*^*KO*^ mice, although it did not improve locomotor rhythmicity, it restored normal levels of activity and body weight as well as longevity (McDearmon et al. 2006) suggesting an important role of muscle-specific *Bmal1* in skeletal muscle maintenance and ageing. In contrast to whole-body *Bmal1*^*KO*^ mice, locomotor activity was normal and there was no sign of sarcomere alterations in muscle-specific *Bmal1*^*KO*^ mice (Dyar et al. 2015; Hodge et al. 2015). However, muscle-specific loss of *Bmal1* resulted in decreased skeletal muscle glucose uptake, glucose oxidation and insulin sensitivity, and coupled with a shift towards more oxidative fibres in the *Soleus* muscle (Dyar et al. 2014). Furthermore, recent data show that similar to global *Bmal1*^*KO*^ mice, muscle-specific loss of *Bmal1* leads to significant changes in bone and cartilage throughout the body, thus implicating the role for skeletal muscle clocks in systemic regulation of peripheral clocks beyond muscle (Schroder et al. 2015). All together, these data indicate a critical role for intrinsic skeletal muscle clocks in regulating local skeletal muscle maintenance during ageing as well as systemic metabolic health.

### Circadian rhythms in skeletal muscle responses to exercise

Recent studies have demonstrated that the molecular clock in skeletal muscle can be dissociated from the rhythm of the SCN by restricting time of feeding or exercise cues (Damiola et al. 2000; Schroder and Esser 2013). Zambon et al. (2003) reported that there is an interaction between time of day and contraction, on the expression of clock genes in human *Quadriceps* muscle after an acute bout of resistance exercise. This suggests that contractile activity might be a zeitgeber for the molecular clock mechanism in skeletal muscle. This was supported by real-time bioluminescence imaging of skeletal muscle tissues from *Per2*::luc luciferase reporter mice following scheduled bouts of either voluntary or involuntary endurance exercise, which showed phase-shifted rhythms in skeletal muscle tissues, but not in the SCN (Wolff and Esser 2012). In addition, voluntary wheel running in arrhythmic clock mutant mice was able to partially rescue the metabolic phenotype of the skeletal muscle (Pastore and Hood 2013). Given that the prognosis of many age-related diseases is strongly linked to the skeletal muscle function (Kadar et al. 2000), these findings illustrate the potential impact of physical activity as an entrainment cue for skeletal muscle clocks.

The time of day has long been known to be important regarding skeletal muscle performances. In humans, more than 20 studies have shown that skeletal muscle torque, strength and power is higher in the late afternoon, between 16:00 and 18:00 h, compared to the morning (Sedliak et al. 2009; Zhang et al. 2009). In addition, a recent study in humans has demonstrated that the individual chronotype is an important parameter in the time of optimal performance (Facer-Childs and Brandstaetter 2015). This concept points out the importance of chronobiology in skeletal muscle activity. Another recent study has reported the influence of daytime scheduled exercise on the expression of genes in schedule-trained horses (McGivney et al. 2010). Interestingly, the peak of the clock-regulated protein UCP3 (Uncoupling Protein 3) preceded the scheduled time of exercise, indicative of an anticipated antioxidant response to ROS during exercise (Murphy et al. 2014). This suggests that the controlled timing of exercise-induced ROS and oxidative damage in tissues with synchronised clock may facilitate downstream redox signalling and repair pathways post-exercise, thus modulating muscle adaptive responses. Indeed, exercising at the ‘right’ time is not only essential for optimal improvement of skeletal muscle performance but also to simultaneously limit damage. In addition to established endogenous sources of DNA damage, night shift work/activity, known to disrupt circadian rhythms in humans, has recently been classified by WHO as a probable carcinogen (class 2a) (Stevens and Zhu 2015).

### Circadian regulation of redox-sensitive pathways and implications for skeletal muscle homoeostasis

Recent studies have demonstrated oscillations in several redox processes including levels of NADP/NADPH (Stringari et al. 2015; Zhou et al. 2015), protein glutathionylation, FAD/NADPH, protein carbonylation and total GSH (Desvergne et al. 2014; Pekovic-Vaughan et al. 2014) as well as oxidation state of peroxiredoxin (Prx) proteins (Edgar et al. 2012; O’Neill and Reddy 2011) and cytosolic release of mitochondrial H_2_O_2_ production (Kil et al. 2015). Consistent with these findings, a wide array of genes involved in the antioxidant response exhibits diurnal oscillations in different species (Beaver et al. 2012; Lai et al. 2012). Moreover, clock mutant mouse models exhibit several ROS-associated phenotypes (Geyfman et al. 2012; Lee et al. 2013; Musiek et al. 2013; Pekovic-Vaughan et al. 2014). Most pronounced is the premature ageing phenotype and shortened lifespan of *Bmal1*^*KO*^ mice, which can be partially rescued by life-long administration of the glutathione (GSH) precursor, *N*-acetyl cysteine (NAC) (Kondratov et al. 2006, 2009). *Bmal1*^*KO*^ mice exhibit high ROS levels and stress-induced premature senescence (Khapre et al. 2011; Lee et al. 2013). We have recently demonstrated that circadian regulation of antioxidant transcription factor NRF2 is required for rhythmic GSH-based antioxidant protection of tissues to oxidative injury through transcriptional regulation of genes involved in the rate-limiting production and utilisation of GSH (Pekovic-Vaughan et al. 2014). Consistent with these findings, *Clock*^*Δ19*^ mutant mice demonstrate decreased levels and activity of NRF2 and total GSH together with increased protein carbonylation, a marker of oxidative protein load (Pekovic-Vaughan et al. 2014). Circadian oscillations of Prx oxidation (Edgar et al. 2012; O’Neill and Reddy 2011), cytosolic H_2_O_2_ and GSH/GSSG ratio (Radha et al. 1985), however, can occur in the absence of transcriptional cycles (Putker and O’Neill 2016), implicating a role for both transcriptional and transcription-independent mechanisms of circadian redox control (Patel et al. 2014). This suggests that the oxidation status of many other important redox-regulated protein thiols might oscillate and thus influence the downstream redox relay signalling pathways. Future research is needed to determine the redox mechanisms of molecular clock function in skeletal muscle, identify the redox relay pathways regulated by the molecular clock and their role in skeletal muscle adaptive responses to exercise and ageing.

## Conclusions

The endogenous redox signals generated by contracting skeletal muscle can activate a number of transcription factors with subsequent effects on gene expression and activation of specific cellular pathways. In young adults, adaptation and rebuilding of skeletal muscle fibres occurs in response to exercise, whilst muscles of older adults have a blunted response to exercise, often associated with dampened or chronic activation of specific transcription factors. The long-term effects of altered cellular signalling and an inability to efficiently rebuild and repair muscle fibres manifests physiologically as a decrease in the number of fibres and atrophy of the remaining fibres. Key transcription factors associated with exercise are regulated by the circadian clock and correct timing of activities could maximise the beneficial effects of exercise, although the circadian clock itself can dampen and/or phase shift during ageing (Yamazaki et al. 2002). Moreover, novel epigenetic regulators, such as miRNAs, add a new layer of redox balance regulation through controlling the expression and activity of transcription factors and enzymes associated with redox signalling. As a better understanding of the canonical mechanisms associated with muscle wasting during ageing is beginning to emerge, and considering the challenges of targeting disrupted redox homoeostasis by antioxidant interventions, novel strategies, such as circadian and microRNA regulation of redox signalling, may be better poised at improving loss of muscle mass and function during ageing.

In skeletal muscle, synchronisation of exercise with circadian clock would ensure optimal activation of signalling pathways for maintaining muscle mass and function (Wolff and Esser 2012). Mimicking the gene expression profile induced as a result of exercise or pharmaceutically through genetic manipulation has also been proposed as a potential therapeutic approach against sarcopenia. Manipulation of miRNA expression represents a potentially powerful therapeutic approach for treating muscle disorders including sarcopenia. Cholesterol-modified microRNA mimics and antagomiRs show efficient delivery in vivo not associated with toxicity (Krutzfeldt et al. 2005). Moreover, miRNA-based therapeutics against liver cancer are currently in clinical trials (http://www.mirnarx.com/pipeline/mirna-MRX34.html). Despite emerging data demonstrating the involvement of miRNAs in regulating skeletal muscle and satellite cell function (Cheung et al. 2012; Soriano-Arroquia et al. 2016a, b), it remains to be established which miRNAs play the key role in regulating sarcopenia development and which may act as a compensatory mechanism. A clue may exist in studies investigating changes in miRNA expression following exercise although, future studies will need to focus on defining the signatures of human sarcopenia and characterising the miRNA:target interactions in muscles in vivo during ageing. Therefore effective miRNA delivery strategies, combined with appropriate circadian delivery time and as well as scheduled exercise interventions based on the chronobiology of skeletal muscle, constitute novel therapeutic approaches for the treatment of age-related muscle wasting and thus warrant further investigation.

## References

[CR1] Adinolfi AM, Yamuy J, Morales FR, Chase MH (1991). Segmental demyelination in peripheral nerves of old cats. Neurobiol Aging.

[CR2] Alvarez-Saavedra M, Antoun G, Yanagiya A, Oliva-Hernandez R, Cornejo-Palma D, Perez-Iratxeta C, Sonenberg N, Cheng HY (2011). miRNA-132 orchestrates chromatin remodeling and translational control of the circadian clock. Hum Mol Genet.

[CR3] Andrews JL, Zhang X, McCarthy JJ, McDearmon EL, Hornberger TA, Russell B, Campbell KS, Arbogast S, Reid MB, Walker JR, Hogenesch JB, Takahashi JS, Esser KA (2010). CLOCK and BMAL1 regulate MyoD and are necessary for maintenance of skeletal muscle phenotype and function. Proc Natl Acad Sci USA.

[CR4] Aoi W, Naito Y, Yoshikawa T (2013). Role of oxidative stress in impaired insulin signaling associated with exercise-induced muscle damage. Free Radic Biol Med.

[CR5] Ardite E, Perdiguero E, Vidal B, Gutarra S, Serrano AL, Munoz-Canoves P (2012). PAI-1-regulated miR-21 defines a novel age-associated fibrogenic pathway in muscular dystrophy. J Cell Biol.

[CR6] Bae K, Lee K, Seo Y, Lee H, Kim D, Choi I (2006). Differential effects of two period genes on the physiology and proteomic profiles of mouse anterior tibialis muscles. Mol Cells.

[CR200] Baird NA, Douglas PM, Simic MS, Grant AR, Moresco JJ, Wolff SC, Yates JR, Manning G, Dillin A (2014). HSF-1-mediated cytoskeletal integrity determines thermotolerance and life span. Science.

[CR201] Baresic M, Salatino S, Kupr B, van Nimwegen E, Handschin C (2014). Transcriptional network analysis in muscle reveals AP-1 as a partner of PGC-1alpha in the regulation of the hypoxic gene program. Mol Cell Biol.

[CR7] Beaver LM, Klichko VI, Chow ES, Kotwica-Rolinska J, Williamson M, Orr WC, Radyuk SN, Giebultowicz JM (2012). Circadian regulation of glutathione levels and biosynthesis in *Drosophila melanogaster*. PLoS ONE.

[CR8] Bernet JD, Doles JD, Hall JK, Kelly Tanaka K, Carter TA, Olwin BB (2014). p38 MAPK signaling underlies a cell-autonomous loss of stem cell self-renewal in skeletal muscle of aged mice. Nat Med.

[CR9] Bhamra I, Compagnone-Post P, O’Neil IA, Iwanejko LA, Bates AD, Cosstick R (2012). Base-pairing preferences, physicochemical properties and mutational behaviour of the DNA lesion 8-nitroguanine. Nucleic Acids Res.

[CR10] Boominathan L (2010). The guardians of the genome (p53, TA-p73, and TA-p63) are regulators of tumor suppressor miRNAs network. Cancer Metastasis Rev.

[CR202] Bori Z, Zhao Z, Koltai E, Fatouros IG, Jamurtas AZ, Douroudos II, Terzis G, Chatzinikolaou A, Sovatzidis A, Draganidis D, Boldogh I, Radak Z (2012). The effects of aging, physical training, and a single bout of exercise on mitochondrial protein expression in human skeletal muscle. Exp Gerontol.

[CR11] Brack AS, Rando TA (2007). Intrinsic changes and extrinsic influences of myogenic stem cell function during aging. Stem Cell Rev.

[CR12] Bregeon D, Peignon PA, Sarasin A (2009). Transcriptional mutagenesis induced by 8-oxoguanine in mammalian cells. PLoS Genet.

[CR13] Brown DM, Goljanek-Whysall K (2015). microRNAs: modulators of the underlying pathophysiology of sarcopenia?. Ageing Res Rev.

[CR14] Brown WF, Strong MJ, Snow R (1988). Methods for estimating numbers of motor units in biceps-brachialis muscles and losses of motor units with aging. Muscle Nerve.

[CR15] Burke LM, Collier GR, Davis PG, Fricker PA, Sanigorski AJ, Hargreaves M (1996). Muscle glycogen storage after prolonged exercise: effect of the frequency of carbohydrate feedings. Am J Clin Nutr.

[CR16] Burkhalter MD, Rudolph KL, Sperka T (2015). Genome instability of ageing stem cells—induction and defence mechanisms. Ageing Res Rev.

[CR17] Butikofer L, Zurlinden A, Bolliger MF, Kunz B, Sonderegger P (2011). Destabilization of the neuromuscular junction by proteolytic cleavage of agrin results in precocious sarcopenia. FASEB J.

[CR18] Caglayan M, Wilson SH (2015). Oxidant and environmental toxicant-induced effects compromise DNA ligation during base excision DNA repair. DNA Repair.

[CR19] Calabrese V, Sultana R, Scapagnini G, Guagliano E, Sapienza M, Bella R, Kanski J, Pennisi G, Mancuso C, Stella AM, Butterfield DA (2006). Nitrosative stress, cellular stress response, and thiol homeostasis in patients with Alzheimer’s disease. Antioxid Redox Signal.

[CR20] Campbell MJ, McComas AJ, Petito F (1973). Physiological changes in ageing muscles. J Neurol Neurosurg Psychiatry.

[CR21] Carlson ME, Suetta C, Conboy MJ, Aagaard P, Mackey A, Kjaer M, Conboy I (2009). Molecular aging and rejuvenation of human muscle stem cells. EMBO Mol Med.

[CR22] Carnio S, LoVerso F, Baraibar MA, Longa E, Khan MM, Maffei M, Reischl M, Canepari M, Loefler S, Kern H, Blaauw B, Friguet B, Bottinelli R, Rudolf R, Sandri M (2014). Autophagy impairment in muscle induces neuromuscular junction degeneration and precocious aging. Cell Rep.

[CR23] Charge SB, Rudnicki MA (2004). Cellular and molecular regulation of muscle regeneration. Physiol Rev.

[CR24] Chatterjee M, Andrulis M, Stuhmer T, Muller E, Hofmann C, Steinbrunn T, Heimberger T, Schraud H, Kressmann S, Einsele H, Bargou RC (2013). The PI3 K/Akt signaling pathway regulates the expression of Hsp70, which critically contributes to Hsp90-chaperone function and tumor cell survival in multiple myeloma. Haematologica.

[CR25] Cheng KC, Cahill DS, Kasai H, Nishimura S, Loeb LA (1992). 8-Hydroxyguanine, an abundant form of oxidative DNA damage, causes G–T and A–C substitutions. J Biol Chem.

[CR26] Cheng HY, Papp JW, Varlamova O, Dziema H, Russell B, Curfman JP, Nakazawa T, Shimizu K, Okamura H, Impey S, Obrietan K (2007). microRNA modulation of circadian-clock period and entrainment. Neuron.

[CR27] Cheung TH, Quach NL, Charville GW, Liu L, Park L, Edalati A, Yoo B, Hoang P, Rando TA (2012). Maintenance of muscle stem-cell quiescence by microRNA-489. Nature.

[CR28] Chu TH, Guo A, Wu W (2014). Down-regulation of apurinic/apyrimidinic endonuclease 1 (APE1) in spinal motor neurones under oxidative stress. Neuropathol Appl Neurobiol.

[CR29] Clark DW, Phang T, Edwards MG, Geraci MW, Gillespie MN (2012). Promoter G-quadruplex sequences are targets for base oxidation and strand cleavage during hypoxia-induced transcription. Free Radic Biol Med.

[CR30] Cobley JN, Bartlett JD, Kayani A, Murray SW, Louhelainen J, Donovan T, Waldron S, Gregson W, Burniston JG, Morton JP, Close GL (2012). PGC-1alpha transcriptional response and mitochondrial adaptation to acute exercise is maintained in skeletal muscle of sedentary elderly males. Biogerontology.

[CR31] Collins CA, Zammit PS, Ruiz AP, Morgan JE, Partridge TA (2007). A population of myogenic stem cells that survives skeletal muscle aging. Stem Cells.

[CR32] Cousin W, Ho ML, Desai R, Tham A, Chen RY, Kung S, Elabd C, Conboy IM (2013). Regenerative capacity of old muscle stem cells declines without significant accumulation of DNA damage. PLoS ONE.

[CR33] Damiola F, Le Minh N, Preitner N, Kornmann B, Fleury-Olela F, Schibler U (2000). Restricted feeding uncouples circadian oscillators in peripheral tissues from the central pacemaker in the suprachiasmatic nucleus. Genes Dev.

[CR203] Dedkov EI, Kostrominova TY, Borisov AB, Carlson BM (2003). MyoD and myogenin protein expression in skeletal muscles of senile rats. Cell and tissue research.

[CR34] Delbono O (2003). Neural control of aging skeletal muscle. Aging Cell.

[CR35] Desvergne A, Ugarte N, Petropoulos I, Friguet B (2014). Circadian modulation of proteasome activities and removal of carbonylated proteins. Free Radic Biol Med.

[CR36] Dibner C, Schibler U, Albrecht U (2010). The mammalian circadian timing system: organization and coordination of central and peripheral clocks. Annu Rev Physiol.

[CR37] Drummond MJ, Dreyer HC, Pennings B, Fry CS, Dhanani S, Dillon EL, Sheffield-Moore M, Volpi E, Rasmussen BB (2008). Skeletal muscle protein anabolic response to resistance exercise and essential amino acids is delayed with aging. J Appl Physiol.

[CR38] Dyar KA, Ciciliot S, Wright LE, Bienso RS, Tagliazucchi GM, Patel VR, Forcato M, Paz MI, Gudiksen A, Solagna F, Albiero M, Moretti I, Eckel-Mahan KL, Baldi P, Sassone-Corsi P, Rizzuto R, Bicciato S, Pilegaard H, Blaauw B, Schiaffino S (2014). Muscle insulin sensitivity and glucose metabolism are controlled by the intrinsic muscle clock. Mol Metab.

[CR39] Dyar KA, Ciciliot S, Tagliazucchi GM, Pallafacchina G, Tothova J, Argentini C, Agatea L, Abraham R, Ahdesmaki M, Forcato M, Bicciato S, Schiaffino S, Blaauw B (2015). The calcineurin-NFAT pathway controls activity-dependent circadian gene expression in slow skeletal muscle. Mol Metab.

[CR40] Edgar RS, Green EW, Zhao Y, van Ooijen G, Olmedo M, Qin X, Xu Y, Pan M, Valekunja UK, Feeney KA, Maywood ES, Hastings MH, Baliga NS, Merrow M, Millar AJ, Johnson CH, Kyriacou CP, O’Neill JS, Reddy AB (2012). Peroxiredoxins are conserved markers of circadian rhythms. Nature.

[CR41] Egan B, Zierath JR (2013). Exercise metabolism and the molecular regulation of skeletal muscle adaptation. Cell Metab.

[CR42] Einsiedel LJ, Luff AR (1992). Alterations in the contractile properties of motor units within the ageing rat medial gastrocnemius. J Neurol Sci.

[CR43] Eisenberg I, Eran A, Nishino I, Moggio M, Lamperti C, Amato AA, Lidov HG, Kang PB, North KN, Mitrani-Rosenbaum S, Flanigan KM, Neely LA, Whitney D, Beggs AH, Kohane IS, Kunkel LM (2007). Distinctive patterns of microRNA expression in primary muscular disorders. Proc Natl Acad Sci USA.

[CR44] Elia L, Quintavalle M, Zhang J, Contu R, Cossu L, Latronico MV, Peterson KL, Indolfi C, Catalucci D, Chen J, Courtneidge SA, Condorelli G (2009). The knockout of miR-143 and -145 alters smooth muscle cell maintenance and vascular homeostasis in mice: correlates with human disease. Cell Death Differ.

[CR45] Facer-Childs E, Brandstaetter R (2015). Circadian phenotype composition is a major predictor of diurnal physical performance in teams. Front Neurol.

[CR46] Friedman RC, Farh KK, Burge CB, Bartel DP (2009). Most mammalian mRNAs are conserved targets of microRNAs. Genome Res.

[CR47] Fry CS, Lee JD, Mula J, Kirby TJ, Jackson JR, Liu F, Yang L, Mendias CL, Dupont-Versteegden EE, McCarthy JJ, Peterson CA (2015). Inducible depletion of satellite cells in adult, sedentary mice impairs muscle regenerative capacity without affecting sarcopenia. Nat Med.

[CR204] Furuyama T, Yamashita H, Kitayama K, Higami Y, Shimokawa I, Mori N (2002). Effects of aging and caloric restriction on the gene expression of Foxo1, 3, and 4 (FKHR, FKHRL1, and AFX) in the rat skeletal muscles. Microsc Res Tech.

[CR48] Gebel S, Lichtner RB, Frushour B, Schlage WK, Hoang V, Talikka M, Hengstermann A, Mathis C, Veljkovic E, Peck M, Peitsch MC, Deehan R, Hoeng J, Westra JW (2013). Construction of a computable network model for DNA damage, autophagy, cell death, and senescence. Bioinform Biol Insights.

[CR206] Gerhart-Hines Z, Rodgers JT, Bare O, Lerin C, Kim SH, Mostoslavsky R, Alt FW, Wu Z, Puigserver P (2007). Metabolic control of muscle mitochondrial function and fatty acid oxidation through SIRT1/PGC-1alpha. EMBO J.

[CR49] Geyfman M, Kumar V, Liu Q, Ruiz R, Gordon W, Espitia F, Cam E, Millar SE, Smyth P, Ihler A, Takahashi JS, Andersen B (2012). Brain and muscle Arnt-like protein-1 (BMAL1) controls circadian cell proliferation and susceptibility to UVB-induced DNA damage in the epidermis. Proc Natl Acad Sci USA.

[CR50] Gianni P, Jan KJ, Douglas MJ, Stuart PM, Tarnopolsky MA (2004). Oxidative stress and the mitochondrial theory of aging in human skeletal muscle. Exp Gerontol.

[CR51] Gil-del Valle L, de la C Milian L, Toledo A, Vilaro N, Otero MA, Tapanes R (2005). Altered redox status in patients with diabetes mellitus type I. Pharmacol Res.

[CR52] Goljanek-Whysall K, Sweetman D, Munsterberg AE (2012). microRNAs in skeletal muscle differentiation and disease. Clin Sci.

[CR53] Gomes AP, Price NL, Ling AJ, Moslehi JJ, Montgomery MK, Rajman L, White JP, Teodoro JS, Wrann CD, Hubbard BP, Mercken EM, Palmeira CM, de Cabo R, Rolo AP, Turner N, Bell EL, Sinclair DA (2013). Declining NAD(+) induces a pseudohypoxic state disrupting nuclear-mitochondrial communication during aging. Cell.

[CR54] Gossan N, Zeef L, Hensman J, Hughes A, Bateman JF, Rowley L, Little CB, Piggins HD, Rattray M, Boot-Handford RP, Meng QJ (2013). The circadian clock in murine chondrocytes regulates genes controlling key aspects of cartilage homeostasis. Arthritis Rheum.

[CR205] Garcia-Prat L, Munoz-Canoves P, Martinez-Vicente M (2016). Dysfunctional autophagy is a driver of muscle stem cell functional decline with aging. Autophagy.

[CR55] Grover-Johnson N, Spencer PS (1981). Peripheral nerve abnormalities in aging rats. J Neuropathol Exp Neurol.

[CR56] Hailer-Morrison MK, Kotler JM, Martin BD, Sugden KD (2003). Oxidized guanine lesions as modulators of gene transcription. Altered p50 binding affinity and repair shielding by 7,8-dihydro-8-oxo-2′-deoxyguanosine lesions in the NF-kappaB promoter element. Biochemistry.

[CR57] Hansen KF, Sakamoto K, Obrietan K (2011). MicroRNAs: a potential interface between the circadian clock and human health. Genome Med.

[CR59] Hastings MH, Reddy AB, Maywood ES (2003). A clockwork web: circadian timing in brain and periphery, in health and disease. Nat Rev Neurosci.

[CR60] He C, Bassik MC, Moresi V, Sun K, Wei Y, Zou Z, An Z, Loh J, Fisher J, Sun Q, Korsmeyer S, Packer M, May HI, Hill JA, Virgin HW, Gilpin C, Xiao G, Bassel-Duby R, Scherer PE, Levine B (2012). Exercise-induced BCL2-regulated autophagy is required for muscle glucose homeostasis. Nature.

[CR61] Hettwer S, Lin S, Kucsera S, Haubitz M, Oliveri F, Fariello RG, Ruegg MA, Vrijbloed JW (2014). Injection of a soluble fragment of neural agrin (NT-1654) considerably improves the muscle pathology caused by the disassembly of the neuromuscular junction. PLoS ONE.

[CR62] Hodge BA, Wen Y, Riley LA, Zhang X, England JH, Harfmann BD, Schroder EA, Esser KA (2015). The endogenous molecular clock orchestrates the temporal separation of substrate metabolism in skeletal muscle. Skelet Muscle.

[CR207] Hollander J, Bejma J, Ookawara T, Ohno H, Ji LL (2000). Superoxide dismutase gene expression in skeletal muscle: fiber-specific effect of age. Mech Ageing Dev.

[CR63] Hu H, Gatti RA (2011). MicroRNAs: new players in the DNA damage response. J Mol Cell Biol.

[CR64] Hu Z, Klein JD, Mitch WE, Zhang L, Martinez I, Wang XH (2014). MicroRNA-29 induces cellular senescence in aging muscle through multiple signaling pathways. Aging.

[CR65] Iyama T, Wilson DM (2013). DNA repair mechanisms in dividing and non-dividing cells. DNA Repair.

[CR66] Jarvis RM, Hughes SM, Ledgerwood EC (2012). Peroxiredoxin 1 functions as a signal peroxidase to receive, transduce, and transmit peroxide signals in mammalian cells. Free Radic Biol Med.

[CR67] Jejurikar SS, Henkelman EA, Cederna PS, Marcelo CL, Urbanchek MG, Kuzon WM (2006). Aging increases the susceptibility of skeletal muscle derived satellite cells to apoptosis. Exp Gerontol.

[CR68] Jeong K, He B, Nohara K, Park N, Shin Y, Kim S, Shimomura K, Koike N, Yoo SH, Chen Z (2015). Dual attenuation of proteasomal and autophagic BMAL1 degradation in Clock Delta19/+ mice contributes to improved glucose homeostasis. Sci Rep.

[CR69] Ji LL, Gomez-Cabrera MC, Steinhafel N, Vina J (2004). Acute exercise activates nuclear factor (NF)-kappaB signaling pathway in rat skeletal muscle. FASEB J.

[CR70] Kadar L, Albertsson M, Areberg J, Landberg T, Mattsson S (2000). The prognostic value of body protein in patients with lung cancer. Ann N Y Acad Sci.

[CR71] Kadi F, Charifi N, Henriksson J (2006). The number of satellite cells in slow and fast fibres from human vastus lateralis muscle. Histochem Cell Biol.

[CR72] Khapre RV, Kondratova AA, Susova O, Kondratov RV (2011). Circadian clock protein BMAL1 regulates cellular senescence in vivo. Cell Cycle.

[CR73] Kil IS, Ryu KW, Lee SK, Kim JY, Chu SY, Kim JH, Park S, Rhee SG (2015). Circadian oscillation of sulfiredoxin in the mitochondria. Mol Cell.

[CR74] Ko CH, Takahashi JS (2006) Molecular components of the mammalian circadian clock. Hum Mol Genet 15 Spec No 2:R271–R277 10.1093/hmg/ddl207 16987893

[CR75] Kojima S, Shingle DL, Green CB (2011). Post-transcriptional control of circadian rhythms. J Cell Sci.

[CR76] Kondratov RV, Kondratova AA, Gorbacheva VY, Vykhovanets OV, Antoch MP (2006). Early aging and age-related pathologies in mice deficient in BMAL1, the core component of the circadian clock. Genes Dev.

[CR77] Kondratov RV, Vykhovanets O, Kondratova AA, Antoch MP (2009). Antioxidant *N*-acetyl-l-cysteine ameliorates symptoms of premature aging associated with the deficiency of the circadian protein BMAL1. Aging (Albany NY).

[CR78] Krutzfeldt J, Rajewsky N, Braich R, Rajeev KG, Tuschl T, Manoharan M, Stoffel M (2005). Silencing of microRNAs in vivo with ‘antagomirs’. Nature.

[CR79] Kuraoka I, Endou M, Yamaguchi Y, Wada T, Handa H, Tanaka K (2003). Effects of endogenous DNA base lesions on transcription elongation by mammalian RNA polymerase II. Implications for transcription-coupled DNA repair and transcriptional mutagenesis. J Biol Chem.

[CR80] Labunskyy VM, Gladyshev VN (2012). Role of reactive oxygen species-mediated signaling in aging. Antioxid Redox Signal.

[CR81] Lai AG, Doherty CJ, Mueller-Roeber B, Kay SA, Schippers JH, Dijkwel PP (2012). CIRCADIAN CLOCK-ASSOCIATED 1 regulates ROS homeostasis and oxidative stress responses. Proc Natl Acad Sci USA.

[CR82] Lal A, Navarro F, Maher CA, Maliszewski LE, Yan N, O’Day E, Chowdhury D, Dykxhoorn DM, Tsai P, Hofmann O, Becker KG, Gorospe M, Hide W, Lieberman J (2009). miR-24 Inhibits cell proliferation by targeting E2F2, MYC, and other cell-cycle genes via binding to “seedless” 3′UTR microRNA recognition elements. Mol Cell.

[CR208] Larsson NG, Wang J, Wilhelmsson H, Oldfors A, Rustin P, Lewandoski M, Barsh GS, Clayton DA (1998). Mitochondrial transcription factor A is necessary for mtDNA maintenance and embryogenesis in mice. Nat Genet.

[CR83] Lapierre LR, Kumsta C, Sandri M, Ballabio A, Hansen M (2015). Transcriptional and epigenetic regulation of autophagy in aging. Autophagy.

[CR84] Larsson L, Ansved T (1995). Effects of ageing on the motor unit. Prog Neurobiol.

[CR85] Lee C, Etchegaray JP, Cagampang FR, Loudon AS, Reppert SM (2001). Posttranslational mechanisms regulate the mammalian circadian clock. Cell.

[CR209] Lee D, Goldberg AL (2015). Muscle wasting in fasting requires activation of NF-kappaB and inhibition of AKT/mechanistic target of rapamycin (mTOR) by the protein acetylase, GCN5. J Biol Chem.

[CR86] Lee J, Moulik M, Fang Z, Saha P, Zou F, Xu Y, Nelson DL, Ma K, Moore DD, Yechoor VK (2013). Bmal1 and beta-cell clock are required for adaptation to circadian disruption, and their loss of function leads to oxidative stress-induced beta-cell failure in mice. Mol Cell Biol.

[CR87] Lexell J, Downham D, Sjostrom M (1986). Distribution of different fibre types in human skeletal muscles. Fibre type arrangement in m. vastus lateralis from three groups of healthy men between 15 and 83 years. J Neurol Sci.

[CR88] Lexell J, Taylor CC, Sjostrom M (1988). What is the cause of the ageing atrophy? Total number, size and proportion of different fiber types studied in whole vastus lateralis muscle from 15- to 83-year-old men. J Neurol Sci.

[CR89] Li MD, Ruan HB, Hughes ME, Lee JS, Singh JP, Jones SP, Nitabach MN, Yang X (2013). O-GlcNAc signaling entrains the circadian clock by inhibiting BMAL1/CLOCK ubiquitination. Cell Metab.

[CR90] Ling SM, Conwit RA, Ferrucci L, Metter EJ (2009). Age-associated changes in motor unit physiology: observations from the Baltimore Longitudinal Study of Aging. Arch Phys Med Rehabil.

[CR91] Liu C, Li S, Liu T, Borjigin J, Lin JD (2007). Transcriptional coactivator PGC-1alpha integrates the mammalian clock and energy metabolism. Nature.

[CR210] Liu X, Manzano G, Lovett DH, Kim HT (2010). Role of AP-1 and RE-1 binding sites in matrix metalloproteinase-2 transcriptional regulation in skeletal muscle atrophy. Biochem Biophys Res Commun.

[CR92] Loudon AS (2012). Circadian biology: a 2.5 billion year old clock. Curr Biol.

[CR93] Marzetti E, Lees HA, Wohlgemuth SE, Leeuwenburgh C (2009). Sarcopenia of aging: underlying cellular mechanisms and protection by calorie restriction. BioFactors.

[CR94] Mates JM, Segura JA, Alonso FJ, Marquez J (2008). Intracellular redox status and oxidative stress: implications for cell proliferation, apoptosis, and carcinogenesis. Arch Toxicol.

[CR95] Mauro A (1961). Satellite cell of skeletal muscle fibers. J Biophys Biochem Cytol.

[CR96] Mayeuf-Louchart A, Staels B, Duez H (2015). Skeletal muscle functions around the clock. Diabetes Obes Metab.

[CR97] McCarthy JJ, Esser KA (2007). MicroRNA-1 and microRNA-133a expression are decreased during skeletal muscle hypertrophy. J Appl Physiol.

[CR98] McCarthy JJ, Andrews JL, McDearmon EL, Campbell KS, Barber BK, Miller BH, Walker JR, Hogenesch JB, Takahashi JS, Esser KA (2007). Identification of the circadian transcriptome in adult mouse skeletal muscle. Physiol Genomics.

[CR99] McCarthy JJ, Mula J, Miyazaki M, Erfani R, Garrison K, Farooqui AB, Srikuea R, Lawson BA, Grimes B, Keller C, Van Zant G, Campbell KS, Esser KA, Dupont-Versteegden EE, Peterson CA (2011). Effective fiber hypertrophy in satellite cell-depleted skeletal muscle. Development.

[CR100] McDearmon EL, Patel KN, Ko CH, Walisser JA, Schook AC, Chong JL, Wilsbacher LD, Song EJ, Hong HK, Bradfield CA, Takahashi JS (2006). Dissecting the functions of the mammalian clock protein BMAL1 by tissue-specific rescue in mice. Science.

[CR101] McDonagh B, Sakellariou GK, Smith NT, Brownridge P, Jackson MJ (2014). Differential cysteine labeling and global label-free proteomics reveals an altered metabolic state in skeletal muscle aging. J Proteome Res.

[CR102] McDonagh B, Scullion SM, Vasilaki A, Pollock N, McArdle A, Jackson MJ (2016). Ageing-induced changes in the redox status of peripheral motor nerves imply an effect on redox signalling rather than oxidative damage. Free Radic Biol Med.

[CR103] McGivney BA, McGettigan PA, Browne JA, Evans AC, Fonseca RG, Loftus BJ, Lohan A, MacHugh DE, Murphy BA, Katz LM, Hill EW (2010). Characterization of the equine skeletal muscle transcriptome identifies novel functional responses to exercise training. BMC Genom.

[CR211] Milan G, Romanello V, Pescatore F, Armani A, Paik JH, Frasson L, Seydel A, Zhao J, Abraham R, Goldberg AL, Blaauw B, DePinho RA, Sandri M (2015). Regulation of autophagy and the ubiquitin-proteasome system by the FoxO transcriptional network during muscle atrophy. Nat Commun.

[CR212] Miller CJ, Gounder SS, Kannan S, Goutam K, Muthusamy VR, Firpo MA, Symons JD, Paine R, Hoidal JR, Rajasekaran NS (2012). Disruption of Nrf2/ARE signaling impairs antioxidant mechanisms and promotes cell degradation pathways in aged skeletal muscle. Biochim Biophys Acta.

[CR104] Miller BH, McDearmon EL, Panda S, Hayes KR, Zhang J, Andrews JL, Antoch MP, Walker JR, Esser KA, Hogenesch JB, Takahashi JS (2007). Circadian and CLOCK-controlled regulation of the mouse transcriptome and cell proliferation. Proc Natl Acad Sci USA.

[CR105] Morgan JE, Partridge TA (2003). Muscle satellite cells. Int J Biochem Cell Biol.

[CR106] Morikawa M, Kino K, Oyoshi T, Suzuki M, Kobayashi T, Miyazawa H (2014). Analysis of guanine oxidation products in double-stranded DNA and proposed guanine oxidation pathways in single-stranded, double-stranded or quadruplex DNA. Biomolecules.

[CR107] Murphy BA, Wagner AL, McGlynn OF, Kharazyan F, Browne JA, Elliott JA (2014). Exercise influences circadian gene expression in equine skeletal muscle. Vet J.

[CR108] Musiek ES, Lim MM, Yang G, Bauer AQ, Qi L, Lee Y, Roh JH, Ortiz-Gonzalez X, Dearborn JT, Culver JP, Herzog ED, Hogenesch JB, Wozniak DF, Dikranian K, Giasson BI, Weaver DR, Holtzman DM, Fitzgerald GA (2013). Circadian clock proteins regulate neuronal redox homeostasis and neurodegeneration. J Clin Invest.

[CR213] Narasimhan M, Hong J, Atieno N, Muthusamy VR, Davidson CJ, Abu-Rmaileh N, Richardson RS, Gomes AV, Hoidal JR, Rajasekaran NS (2014). Nrf2 deficiency promotes apoptosis and impairs PAX7/MyoD expression in aging skeletal muscle cells. Free Radic Biol Med.

[CR109] Nielsen S, Scheele C, Yfanti C, Akerstrom T, Nielsen AR, Pedersen BK, Laye MJ (2010). Muscle specific microRNAs are regulated by endurance exercise in human skeletal muscle. J Physiol.

[CR110] O’Driscoll M, Jeggo PA (2008). CsA can induce DNA double-strand breaks: implications for BMT regimens particularly for individuals with defective DNA repair. Bone Marrow Transplant.

[CR111] Olah G, Szczesny B, Brunyanszki A, Lopez-Garcia IA, Gero D, Radak Z, Szabo C (2015). Differentiation-associated downregulation of poly(ADP-ribose) polymerase-1 expression in myoblasts serves to increase their resistance to oxidative stress. PLoS ONE.

[CR112] Olejniczak SH, La Rocca G, Gruber JJ, Thompson CB (2013). Long-lived microRNA–Argonaute complexes in quiescent cells can be activated to regulate mitogenic responses. Proc Natl Acad Sci USA.

[CR113] O’Neill JS, Reddy AB (2011). Circadian clocks in human red blood cells. Nature.

[CR114] O’Neill HM, Holloway GP, Steinberg GR (2013). AMPK regulation of fatty acid metabolism and mitochondrial biogenesis: implications for obesity. Mol Cell Endocrinol.

[CR115] Pallafacchina G, Francois S, Regnault B, Czarny B, Dive V, Cumano A, Montarras D, Buckingham M (2010). An adult tissue-specific stem cell in its niche: a gene profiling analysis of in vivo quiescent and activated muscle satellite cells. Stem Cell Res.

[CR116] Panda S, Sato TK, Castrucci AM, Rollag MD, DeGrip WJ, Hogenesch JB, Provencio I, Kay SA (2002). Melanopsin (Opn4) requirement for normal light-induced circadian phase shifting. Science.

[CR214] Pardo PS, Lopez MA, Boriek AM (2008). FOXO transcription factors are mechanosensitive and their regulation is altered with aging in the respiratory pump. Am J Physiol Cell Physiol.

[CR117] Pastore S, Hood DA (2013). Endurance training ameliorates the metabolic and performance characteristics of circadian Clock mutant mice. J Appl Physiol.

[CR118] Patel DB, Chauhan KR, Mukhopadhyay I (2014). Unraveling the photoelectrochemical properties of ionic liquids: cognizance of partially reversible redox activity. Phys Chem Chem Phys.

[CR119] Pearson T, Kabayo T, Ng R, Chamberlain J, McArdle A, Jackson MJ (2014). Skeletal muscle contractions induce acute changes in cytosolic superoxide, but slower responses in mitochondrial superoxide and cellular hydrogen peroxide. PLoS ONE.

[CR120] Pekovic-Vaughan V, Gibbs J, Yoshitane H, Yang N, Pathiranage D, Guo B, Sagami A, Taguchi K, Bechtold D, Loudon A, Yamamoto M, Chan J, van der Horst GT, Fukada Y, Meng QJ (2014). The circadian clock regulates rhythmic activation of the NRF2/glutathione-mediated antioxidant defense pathway to modulate pulmonary fibrosis. Genes Dev.

[CR121] Piasecki M, Ireland A, Stashuk D, Hamilton-Wright A, Jones DA, McPhee JS (2015). Age-related neuromuscular changes affecting human vastus lateralis. J Physiol.

[CR215] Picca A, Pesce V, Fracasso F, Joseph AM, Leeuwenburgh C, Lezza AM (2014). A comparison among the tissue-specific effects of aging and calorie restriction on TFAM amount and TFAM-binding activity to mtDNA in rat. Biochim Biophys Acta.

[CR122] Poole LB (2015). The basics of thiols and cysteines in redox biology and chemistry. Free Radic Biol Med.

[CR123] Powers SK, Jackson MJ (2008). Exercise-induced oxidative stress: cellular mechanisms and impact on muscle force production. Physiol Rev.

[CR124] Putker M, O’Neill JS (2016). Reciprocal control of the circadian clock and cellular redox state—a critical appraisal. Mol Cells.

[CR125] Putker M, Vos HR, van Dorenmalen K, de Ruiter H, Duran AG, Snel B, Burgering BM, Vermeulen M, Dansen TB (2015). Evolutionary acquisition of cysteines determines FOXO paralog-specific redox signaling. Antioxid Redox Signal.

[CR126] Radak Z, Bori Z, Koltai E, Fatouros IG, Jamurtas AZ, Douroudos II, Terzis G, Nikolaidis MG, Chatzinikolaou A, Sovatzidis A, Kumagai S, Naito H, Boldogh I (2011). Age-dependent changes in 8-oxoguanine-DNA glycosylase activity are modulated by adaptive responses to physical exercise in human skeletal muscle. Free Radic Biol Med.

[CR127] Radha E, Hill TD, Rao GH, White JG (1985). Glutathione levels in human platelets display a circadian rhythm in vitro. Thromb Res.

[CR128] Reppert SM, Weaver DR (2002). Coordination of circadian timing in mammals. Nature.

[CR129] Rivas DA, Lessard SJ, Rice NP, Lustgarten MS, So K, Goodyear LJ, Parnell LD, Fielding RA (2014). Diminished skeletal muscle microRNA expression with aging is associated with attenuated muscle plasticity and inhibition of IGF-1 signaling. FASEB J.

[CR130] Romanello V, Sandri M (2015). Mitochondrial quality control and muscle mass maintenance. Front Physiol.

[CR216] Safdar A, deBeer J, Tarnopolsky MA (2010). Dysfunctional Nrf2-Keap1 redox signaling in skeletal muscle of the sedentary old. Free Radic Biol Med.

[CR131] Sakellariou GK, Vasilaki A, Palomero J, Kayani A, Zibrik L, McArdle A, Jackson MJ (2013). Studies of mitochondrial and nonmitochondrial sources implicate nicotinamide adenine dinucleotide phosphate oxidase(s) in the increased skeletal muscle superoxide generation that occurs during contractile activity. Antioxid Redox Signal.

[CR132] Sakellariou GK, Davis CS, Shi Y, Ivannikov MV, Zhang Y, Vasilaki A, Macleod GT, Richardson A, Van Remmen H, Jackson MJ, McArdle A, Brooks SV (2014). Neuron-specific expression of CuZnSOD prevents the loss of muscle mass and function that occurs in homozygous CuZnSOD-knockout mice. FASEB J.

[CR133] Sakellariou GK, Jackson MJ, Vasilaki A (2014). Redefining the major contributors to superoxide production in contracting skeletal muscle. The role of NAD(P)H oxidases. Free Radic Res.

[CR217] Sandri M (2010). Autophagy in skeletal muscle. FEBS Lett.

[CR218] Sandri M, Sandri C, Gilbert A, Skurk C, Calabria E, Picard A, Walsh K, Schiaffino S, Lecker SH, Goldberg AL (2004). Foxo transcription factors induce the atrophy-related ubiquitin ligase atrogin-1 and cause skeletal muscle atrophy. Cell.

[CR219] Sartori R, Milan G, Patron M, Mammucari C, Blaauw B, Abraham R, Sandri M (2009). Smad2 and 3 transcription factors control muscle mass in adulthood. Am J Physiol Cell Physiol.

[CR134] Schroder EA, Esser KA (2013). Circadian rhythms, skeletal muscle molecular clocks, and exercise. Exerc Sport Sci Rev.

[CR135] Schroder EA, Harfmann BD, Zhang X, Srikuea R, England JH, Hodge BA, Wen Y, Riley LA, Yu Q, Christie A, Smith JD, Seward T, Wolf Horrell EM, Mula J, Peterson CA, Butterfield TA, Esser KA (2015). Intrinsic muscle clock is necessary for musculoskeletal health. J Physiol.

[CR136] Schroeder AM, Truong D, Loh DH, Jordan MC, Roos KP, Colwell CS (2012). Voluntary scheduled exercise alters diurnal rhythms of behaviour, physiology and gene expression in wild-type and vasoactive intestinal peptide-deficient mice. J Physiol.

[CR137] Sedliak M, Finni T, Cheng S, Lind M, Hakkinen K (2009). Effect of time-of-day-specific strength training on muscular hypertrophy in men. J Strength Cond Res.

[CR138] Settembre C, Di Malta C, Polito VA, Garcia Arencibia M, Vetrini F, Erdin S, Erdin SU, Huynh T, Medina D, Colella P, Sardiello M, Rubinsztein DC, Ballabio A (2011). TFEB links autophagy to lysosomal biogenesis. Science.

[CR139] Sharma AK, Bajada S, Thomas PK (1980). Age changes in the tibial and plantar nerves of the rat. J Anat.

[CR140] Shefer G, Rauner G, Yablonka-Reuveni Z, Benayahu D (2010). Reduced satellite cell numbers and myogenic capacity in aging can be alleviated by endurance exercise. PLoS ONE.

[CR141] Sheng Z, Oka S, Tsuchimoto D, Abolhassani N, Nomaru H, Sakumi K, Yamada H, Nakabeppu Y (2012). 8-Oxoguanine causes neurodegeneration during MUTYH-mediated DNA base excision repair. J Clin Investig.

[CR142] Snijders T, Wall BT, Dirks ML, Senden JM, Hartgens F, Dolmans J, Losen M, Verdijk LB, van Loon LJ (2014). Muscle disuse atrophy is not accompanied by changes in skeletal muscle satellite cell content. Clin Sci.

[CR143] Soares RJ, Cagnin S, Chemello F, Silvestrin M, Musaro A, De Pitta C, Lanfranchi G, Sandri M (2014). Involvement of microRNAs in the regulation of muscle wasting during catabolic conditions. J Biol Chem.

[CR144] Sobotta MC, Liou W, Stocker S, Talwar D, Oehler M, Ruppert T, Scharf AN, Dick TP (2015). Peroxiredoxin-2 and STAT3 form a redox relay for H2O2 signaling. Nat Chem Biol.

[CR145] Soriano-Arroquia A, House L, Tregilgas L, Canty-Laird E, Goljanek-Whysall K (2016a) The functional consequences of age-related changes in microRNA expression in skeletal muscle. Biogerontology 10.1007/s10522-016-9638-8 PMC4889642 26922183

[CR146] Soriano-Arroquia A, McCormick R, Molloy AP, McArdle A, Goljanek-Whysall K (2016b) Age-related changes in miR-143-3p:Igfbp5 interactions affect muscle regeneration. Aging Cell 10.1111/acel.12442 PMC4783349 26762731

[CR147] Sousa-Victor P, Gutarra S, Garcia-Prat L, Rodriguez-Ubreva J, Ortet L, Ruiz-Bonilla V, Jardi M, Ballestar E, Gonzalez S, Serrano AL, Perdiguero E, Munoz-Canoves P (2014). Geriatric muscle stem cells switch reversible quiescence into senescence. Nature.

[CR148] Staiger D, Green R (2011). RNA-based regulation in the plant circadian clock. Trends Plant Sci.

[CR149] Stamler JS, Meissner G (2001). Physiology of nitric oxide in skeletal muscle. Physiol Rev.

[CR150] Steenken S, Jovanovic SV (1997). How easily oxidizable is DNA? One-electron reduction potentials of adenosine and guanosine radicals in aqueous solution. J Am Chem Soc.

[CR151] Stevens RG, Zhu Y (2015). Re: Bracci M et al. “Rotating-shift nurses after a day off: peripheral clock gene expression, urinary melatonin, and serum 17-estradiol levels”. Scand J Work Environ Health.

[CR152] Stringari C, Wang H, Geyfman M, Crosignani V, Kumar V, Takahashi JS, Andersen B, Gratton E (2015). In vivo single-cell detection of metabolic oscillations in stem cells. Cell Rep.

[CR153] Sun QA, Wang B, Miyagi M, Hess DT, Stamler JS (2013). Oxygen-coupled redox regulation of the skeletal muscle ryanodine receptor/Ca^2+^ release channel (RyR1): sites and nature of oxidative modification. J Biol Chem.

[CR154] Suzuki HI, Yamagata K, Sugimoto K, Iwamoto T, Kato S, Miyazono K (2009). Modulation of microRNA processing by p53. Nature.

[CR220] Tang AH, Rando TA (2014). Induction of autophagy supports the bioenergetic demands of quiescent muscle stem cell activation. EMBO J.

[CR155] Togliatto G, Trombetta A, Dentelli P, Cotogni P, Rosso A, Tschop MH, Granata R, Ghigo E, Brizzi MF (2013). Unacylated ghrelin promotes skeletal muscle regeneration following hindlimb ischemia via SOD-2-mediated miR-221/222 expression. J Am Heart Assoc.

[CR156] Tong X, Hou X, Jourd’heuil D, Weisbrod RM, Cohen RA (2010). Upregulation of Nox4 by TGF{beta}1 oxidizes SERCA and inhibits NO in arterial smooth muscle of the prediabetic Zucker rat. Circ Res.

[CR157] Vahidi Ferdousi L, Rocheteau P, Chayot R, Montagne B, Chaker Z, Flamant P, Tajbakhsh S, Ricchetti M (2014). More efficient repair of DNA double-strand breaks in skeletal muscle stem cells compared to their committed progeny. Stem cell research.

[CR158] Vainshtein A, Grumati P, Sandri M, Bonaldo P (2014). Skeletal muscle, autophagy, and physical activity: the menage a trois of metabolic regulation in health and disease. J Mol Med (Berl).

[CR159] Valdez G, Tapia JC, Kang H, Clemenson GD, Gage FH, Lichtman JW, Sanes JR (2010). Attenuation of age-related changes in mouse neuromuscular synapses by caloric restriction and exercise. Proc Natl Acad Sci USA.

[CR160] Valdez G, Heyer MP, Feng G, Sanes JR (2014). The role of muscle microRNAs in repairing the neuromuscular junction. PLoS ONE.

[CR161] Vasilaki A, Mansouri A, Van Remmen H, van der Meulen JH, Larkin L, Richardson AG, McArdle A, Faulkner JA, Jackson MJ (2006). Free radical generation by skeletal muscle of adult and old mice: effect of contractile activity. Aging Cell.

[CR221] Vasilaki A, McArdle F, Iwanejko LM, McArdle A (2006). Adaptive responses of mouse skeletal muscle to contractile activity: the effect of age. Mech Ageing Dev.

[CR162] Vasilaki A, van der Meulen JH, Larkin L, Harrison DC, Pearson T, Van Remmen H, Richardson A, Brooks SV, Jackson MJ, McArdle A (2010). The age-related failure of adaptive responses to contractile activity in skeletal muscle is mimicked in young mice by deletion of Cu, Zn superoxide dismutase. Aging Cell.

[CR163] Verdijk LB, Koopman R, Schaart G, Meijer K, Savelberg HH, van Loon LJ (2007). Satellite cell content is specifically reduced in type II skeletal muscle fibers in the elderly. Am J Physiol Endocrinol Metab.

[CR164] Verdu E, Ceballos D, Vilches JJ, Navarro X (2000). Influence of aging on peripheral nerve function and regeneration. J Peripher Nerv Syst.

[CR165] Vicencio JM, Galluzzi L, Tajeddine N, Ortiz C, Criollo A, Tasdemir E, Morselli E, Ben Younes A, Maiuri MC, Lavandero S, Kroemer G (2008). Senescence, apoptosis or autophagy? When a damaged cell must decide its path–a mini-review. Gerontology.

[CR166] Wang P, Zou F, Zhang X, Li H, Dulak A, Tomko RJ, Lazo JS, Wang Z, Zhang L, Yu J (2009). microRNA-21 negatively regulates Cdc25A and cell cycle progression in colon cancer cells. Cancer Res.

[CR168] Wang JX, Gao J, Ding SL, Wang K, Jiao JQ, Wang Y, Sun T, Zhou LY, Long B, Zhang XJ, Li Q, Liu JP, Feng C, Liu J, Gong Y, Zhou Z, Li PF (2015). Oxidative modification of miR-184 enables it to target Bcl-xL and Bcl-w. Mol Cell.

[CR169] Ward CW, Prosser BL, Lederer WJ (2013). Mechanical stretch induced activation of ROS/RNS signaling in striated muscle. Antioxid Redox Signal.

[CR222] White TA, LeBrasseur NK (2014). Myostatin and sarcopenia: opportunities and challenges—a mini-review. Gerontology.

[CR170] Williams AH, Valdez G, Moresi V, Qi X, McAnally J, Elliott JL, Bassel-Duby R, Sanes JR, Olson EN (2009). MicroRNA-206 delays ALS progression and promotes regeneration of neuromuscular synapses in mice. Science.

[CR171] Woldt E, Sebti Y, Solt LA, Duhem C, Lancel S, Eeckhoute J, Hesselink MK, Paquet C, Delhaye S, Shin Y, Kamenecka TM, Schaart G, Lefebvre P, Neviere R, Burris TP, Schrauwen P, Staels B, Duez H (2013). Rev-erb-alpha modulates skeletal muscle oxidative capacity by regulating mitochondrial biogenesis and autophagy. Nat Med.

[CR172] Wolff G, Esser KA (2012). Scheduled exercise phase shifts the circadian clock in skeletal muscle. Med Sci Sports Exerc.

[CR173] Yamazaki S, Straume M, Tei H, Sakaki Y, Menaker M, Block GD (2002). Effects of aging on central and peripheral mammalian clocks. Proc Natl Acad Sci USA.

[CR174] Yuan J, Tang Z, Yang S, Li K (2013). CRABP2 promotes myoblast differentiation and is modulated by the transcription factors MyoD and Sp1 in C2C12 cells. PLoS ONE.

[CR175] Zambon AC, McDearmon EL, Salomonis N, Vranizan KM, Johansen KL, Adey D, Takahashi JS, Schambelan M, Conklin BR (2003). Time- and exercise-dependent gene regulation in human skeletal muscle. Genome Biol.

[CR176] Zhang EE, Liu AC, Hirota T, Miraglia LJ, Welch G, Pongsawakul PY, Liu X, Atwood A, Huss JW, Janes J, Su AI, Hogenesch JB, Kay SA (2009). A genome-wide RNAi screen for modifiers of the circadian clock in human cells. Cell.

[CR177] Zhang J, Li H, Teng H, Zhang T, Luo Y, Zhao M, Li YQ, Sun ZS (2012). Regulation of peripheral clock to oscillation of substance P contributes to circadian inflammatory pain. Anesthesiology.

[CR178] Zhang Y, Yang L, Gao YF, Fan ZM, Cai XY, Liu MY, Guo XR, Gao CL, Xia ZK (2013). MicroRNA-106b induces mitochondrial dysfunction and insulin resistance in C2C12 myotubes by targeting mitofusin-2. Mol Cell Endocrinol.

[CR179] Zheng B, Larkin DW, Albrecht U, Sun ZS, Sage M, Eichele G, Lee CC, Bradley A (1999). The mPer2 gene encodes a functional component of the mammalian circadian clock. Nature.

[CR180] Zhou M, Wang W, Karapetyan S, Mwimba M, Marques J, Buchler NE, Dong X (2015). Redox rhythm reinforces the circadian clock to gate immune response. Nature.

